# IOBR: Multi-Omics Immuno-Oncology Biological Research to Decode Tumor Microenvironment and Signatures

**DOI:** 10.3389/fimmu.2021.687975

**Published:** 2021-07-02

**Authors:** Dongqiang Zeng, Zilan Ye, Rongfang Shen, Guangchuang Yu, Jiani Wu, Yi Xiong, Rui Zhou, Wenjun Qiu, Na Huang, Li Sun, Xuejun Li, Jianping Bin, Yulin Liao, Min Shi, Wangjun Liao

**Affiliations:** ^1^ Department of Oncology, Nanfang Hospital, Southern Medical University, Guangzhou, China; ^2^ State Key Laboratory of Molecular Oncology, Department of Etiology and Carcinogenesis, National Cancer Center/National Clinical Research Center for Cancer/Cancer Hospital, Chinese Academy of Medical Sciences and Peking Union Medical College, Beijing, China; ^3^ Department of Bioinformatics, School of Basic Medical Sciences, Southern Medical University, Guangzhou, China; ^4^ Department of Neurosurgery, Xiangya Hospital, Central South University, Changsha, China; ^5^ Hunan International Scientific and Technological Cooperation Base of Brain Tumor Research, Xiangya Hospital, Central South University, Changsha, China; ^6^ Xiangya School of Medicine, Central South University, Changsha, China; ^7^ Department of Cardiology, Nanfang Hospital, Southern Medical University, Guangzhou, China

**Keywords:** tumor microenvironment, multi-omics, gene signatures, immune-tumor interaction, metabolism

## Abstract

Recent advances in next-generation sequencing (NGS) technologies have triggered the rapid accumulation of publicly available multi-omics datasets. The application of integrated omics to explore robust signatures for clinical translation is increasingly emphasized, and this is attributed to the clinical success of immune checkpoint blockades in diverse malignancies. However, effective tools for comprehensively interpreting multi-omics data are still warranted to provide increased granularity into the intrinsic mechanism of oncogenesis and immunotherapeutic sensitivity. Therefore, we developed a computational tool for effective Immuno-Oncology Biological Research (IOBR), providing a comprehensive investigation of the estimation of reported or user-built signatures, TME deconvolution, and signature construction based on multi-omics data. Notably, IOBR offers batch analyses of these signatures and their correlations with clinical phenotypes, long non-coding RNA (lncRNA) profiling, genomic characteristics, and signatures generated from single-cell RNA sequencing (scRNA-seq) data in different cancer settings. Additionally, IOBR integrates multiple existing microenvironmental deconvolution methodologies and signature construction tools for convenient comparison and selection. Collectively, IOBR is a user-friendly tool for leveraging multi-omics data to facilitate immuno-oncology exploration and to unveil tumor-immune interactions and accelerating precision immunotherapy.

## Introduction

The clinical success of immune checkpoint blockade (ICB) has recently seen progress due to the immunotherapy that revolutionizes the treatment paradigm of advanced cancers. However, the heterogeneous immunotherapy outcomes across patients necessitate the investigation into host-tumor interactions, particularly the immune cell infiltration within the tumor microenvironment (TME), to define robust predictive biomarkers for precision therapy. In this regard, increasing TME-relevant gene signatures have been reported to estimate immune contexture and predict clinical treatment response. Notably, gene expression profiling (GEP) (1) and TMEscore (2) are influential pan-cancer predictive signatures for prognosis, ICB response, and resistance by decoding the TME component using transcriptomic data. Gene signatures for chemotherapy response prediction have also been reported: the 70-gene (3) and 21-gene (4) assays predict distant recurrence of estrogen receptor positive breast cancer with adjuvant chemotherapy, with the aforementioned TMEscore further promising as a biomarker for chemotherapy sensitivity in late-stage gastric cancer (2). Signatures such as PAM50, constructed by integrating transcriptomics with other omics (genomics, methylation, and proteomics) to define subgroups, provide a new lens into tumor plasticity and heterogeneity of breast cancer (5).

The emergence of these promising signatures is greatly attributed to the development of NGS and computational deconvolution methodology. Technological breakthroughs in NGS have driven an enormous accumulation of publicly available multi-omics datasets, allowing easy accessibility for multi-omics data. Despite the rapid technological progress of scRNA-seq, the lack of large datasets indicates that the validation of signatures still heavily depends on attainable bulk RNA-seq datasets. Additionally, based on transcriptomic data, recently developed computational algorithms and tools were utilized to dissect tumor-TME interactions. Tools for TME deconvolution are fundamentally classified according to four computational principles: machine learning, gene set enrichment analysis (GSEA), linear regression, and nonlinear programming (6). Nonlinear programming-based principles do not necessarily rely on the information of different cell-type frequencies, whereas the other three counterparts require prior knowledge of marker genes of distinct immune cell subsets and molecular profiles (6). Machine learning based principles could evaluate the absolute proportion of infiltrating immune cells within the TME, while gene set enrichment analysis-based principles infer the relative proportion (6).

Given the merits of the aforementioned deconvolution methods, further comparisons of the results for additional accuracy and the subsequent downstream analyses are not covered by either of these tools. Competent tools to conveniently interpret transcriptomic or integrated omics data are warranted to offer new insight into tumorigenesis, immune-tumor interaction, and therapeutic sensitivity diversity. Therefore, we developed a computational tool known as IOBR, to comprehensively explore and visualize the following: multi-omics interpretation, including signature score calculation and systematic estimation of its correlations with clinical phenotypes; noncoding RNA characteristics; signatures derived from scRNA-seq data and genomic landscapes in multiple cancers; as well as TME deconvolution with diverse algorithms and fast signature construction. For a much broader impact and usage of the IOBR tool, we also created an IOBR Shinny application. This application is a user-friendly web-based interface allowing fundamental researchers without skill in R programming to leverage the merits of this multifunctional tool. Together, IOBR is an effective tool, and its implementation in the study of immuno-oncology may aid in the discovery of novel tumor-immune interactions and accelerating precision immunotherapy.

## Materials and Methods

### Data Preprocessing

The multi-omics data retrieved from a trial of atezolizumab for bladder cancer (IMvigor210) (7) were downloaded. Subsequently, we transformed the count matrix into Transcripts Per Kilobase Million (TPM) format by executing *count2tpm* function, and we conducted gene annotation by utilizing *anno_eset* function in IOBR.

### Function Modules and Implementation

IOBR is a user-friendly tool, and the detailed implementation of IOBR was illustrated in the tutorial (https://github.com/IOBR/IOBR) with a complete analysis pipeline. IOBR consists of four functional modules, comprising an estimation of signature scores and signatures generated from scRNA-seq data, along with decoding immune contexture (signature and TME deconvolution module); identification of phenotype relevant signatures, cell fraction, or signature genes, as well as pertinent batch statistical analyses (phenotype module); analysis of signature associated mutations (mutation module) and fast model construction (model construction module).

### Signature and TME Estimation Module

#### Signature Estimation

To elucidate an increasingly granular view of the TME cellular composition and functional status with the goal of cancer-therapy refinement, we constructed an estimation function for user-generated signatures or 255 reported signatures enrolled in IOBR (**Supplementary Table S1**). The extensive signature collection is classified into three categories: TME-associated, tumor-metabolism, and tumor-intrinsic signatures. Additionally, IOBR supports the estimation of the signature gene sets derived from the GO, KEGG, HALLMARK, and REACTOME databases. IOBR permits users to generate a signature list based on their own biological discovery or expletory requirement, for convenient estimation and follow-up systematic exploration. The web-based interface of IOBR also allows researchers to effectively calculate signature scores by setting corresponding parameters.

Three methodologies were included in the process of signature score evaluation, comprising Single-sample Gene Set Enrichment Analysis (ssGSEA), Principal Component Analysis (PCA), and Z-score. ssGSEA is a wildly-adopted tool for calculating separate enrichment scores for each pairing of a sample and gene set (8). Each ssGSEA enrichment score represents the degree to which the genes in a particular gene set are coordinately up- or down-regulated within a sample. Notably, PCA computes the principal components for performing a change of basis on the exploratory data for predictive model construction. Current signatures constructed using PCA methodology include the Pan-F-TBRs (7) and the TMEscore (2), two promising biomarkers for predicting clinical outcomes and therapy sensitivity of malignancies. Z-score is a numerical measurement for describing a score’s relationship to the mean of a group of values. Z-score is measured in terms of standard deviations from the mean. These three methods are able to be selected in IOBR by inputting targeted methods or integrations, with corresponding visualizations supported.

#### Signatures Derived From scRNA-seq Data

The technological and computational innovations of single-cell analysis make it a popular alternative for determining cell markers and gene signatures for phenotypes. However, the significantly expensive cost and high requirement for starting tumor material limits its widespread utility. The large and attainable bulk RNA-seq datasets continue to be the major workhorse for validating the signatures generated from single-cell analysis. Thereafter, IOBR provides multiple methodologies for extracting cell signature genes from scRNA-seq data (TPM or counts matrix are available inputs). Remarkably, the linear Support Vector Regression (SVR) algorithm of CIBERSORT or the LSEI (9) algorithms are implemented in IOBR for convenient bulk RNA-seq data analysis for verifying the clinical value of the targeted cells identified by scRNA-seq data.

#### TME Deconvolution

Clinical investigations have highlighted cell infiltrations in TME as pivotal contributors to the complex anti-tumor immunity in malignancies. TME-cell deconvolution is the major technological hurdle, and the deconvolution algorithms vary in their merits and pitfalls (10, 11). IOBR integrates eight open-source deconvolution methodologies, namely, CIBERSORT (12), ESTIMATE (13), quanTIseq (14), TIMER (15), IPS (16), MCPCounter (17), xCell (18), and EPIC (19).

CIBERSORT is the most well-recognized method for detecting 22 immune cells in TME, allowing large-scale analysis of RNA mixtures for cellular biomarkers and therapeutic targets with promising accuracy (12). Notably, through the adoption of the linear vector regression principle of CIBERSORT, IOBR allows users to construct a self-defined signature. The availability of its input file was extended to cell-subsets derived from single-cell sequencing results. ESTEMATE dissects non-malignant contextures, including stromal and immune signatures, to determine tumor purity (13). The quanTIseq method enumerates 10 immune cell subsets from bulk RNAseq data (14). TIMER quantifies the abundance of six tumor-infiltrating immune compartments and provides six major analytic modules for analyzing the immune infiltration with other cancer molecular profiles (15). IPS estimates 28 TIL subpopulations, including effector and memory T cells and immunosuppressive cells (16). MCP-counter conducts robust quantification of the absolute abundance of eight immune and two stromal cell populations in heterogeneous tissues from transcriptomic data (17). xCell provides a comprehensive view of 64 immune cells from RNA-seq data and other cell subsets in bulk tumor tissue (18). EPIC decodes the proportion of immune and cancer cells from the expression of genes and compares it with the gene expression profiles from specific cells to predict the cell subpopulation landscape (19). In a nutshell, IOBR R package and web-based interface enable the convenient integration and visualization of the above-mentioned deconvolution results and a flexible selection of particular methodologies of interest.

### Phenotype Module

To implement the aforementioned TME deconvolution and signatures calculation for exploring potential clinical translation, we collected and systematically categorized the signatures into 39 groups (**Supplementary Table S2**). The categories involve TME cell populations (classified either by deconvolution methods or cell types), signatures of immunophenotype, tumor metabolism, hypoxia, and EMT. Furthermore, IOBR supports the construction of a novel signature group derived from their own immuno-oncological findings, which lays the foundation for subsequent minding latent biological mechanisms and potential clinical translation.

Collectively, the phenotype module of the IOBR R package permits systematic identification of phenotype relevant signatures, cell fraction, or signature genes, as well as corresponding batch statistical analyses and visualization.

### Mutation Module

In addition to systematical signature-phenotype investigation, IOBR expands the transcriptomic exploration to the interplay within genome profiles. Genome data in Mutation Annotation Format (MAF) format (20) downloaded from the University of California, Santa Cruz (UCSC) website, or user-construct mutation matrices are acceptable as an input to find mutations related to specific signatures. Furthermore, IOBR supports transforming the MAF data into a mutation matrix with distinct variation types comprising insertion–deletion mutations (indel), single-nucleotide polymorphism (SNP), frameshift, or an integration of all of the mutation types for flexible selection. Wilcoxon rank-sum test is employed in this module for batch analysis of mutations significantly associated with targeted signatures. IOBR also supports batch visualization of the mutation statutes (mutation or non-mutation) of interest.

### Model Construction Module

For effective application of the signatures in clinical interpretation, IOBR provides functions for feature selection, robust biomarker identification, and model construction based on prior identified phenotype associated signatures. To our knowledge, the therapeutic response and overall survival are focused endpoints in oncology, and leveraging the corresponding signatures to construct models may hold promise in precise and cost-effective prediction of tumor prognosis and treatment sensitivity. Moreover, rational utility in other bioscience settings may also shed new light on uncovering novel discoveries of interest.

### Software Availability

R package: https://github.com/IOBR/IOBR

The web-based interface: https://yi-xiong.shinyapps.io/IOBRshiny/.

### Data Availability

In a recently published manuscript with multi-omics data retrieved from a trial of atezolizumab for bladder cancer (IMvigor210) (7), we generated immunotherapy associated risk score, determined the TME infiltration pattern, further identified macrophages as a robust predictive biomarker, subsequently unveiled the predominant genomic alterations, and significant metabolic characteristics with the assistance of the IOBR tool (21).

## Results

To comprehensively leverage the transcriptomic data to detect immune-tumor interplay and its promising clinical translation, we introduce the IOBR R package as an effective and flexible tool.

### IOBR Workflow

**Figure 1:**
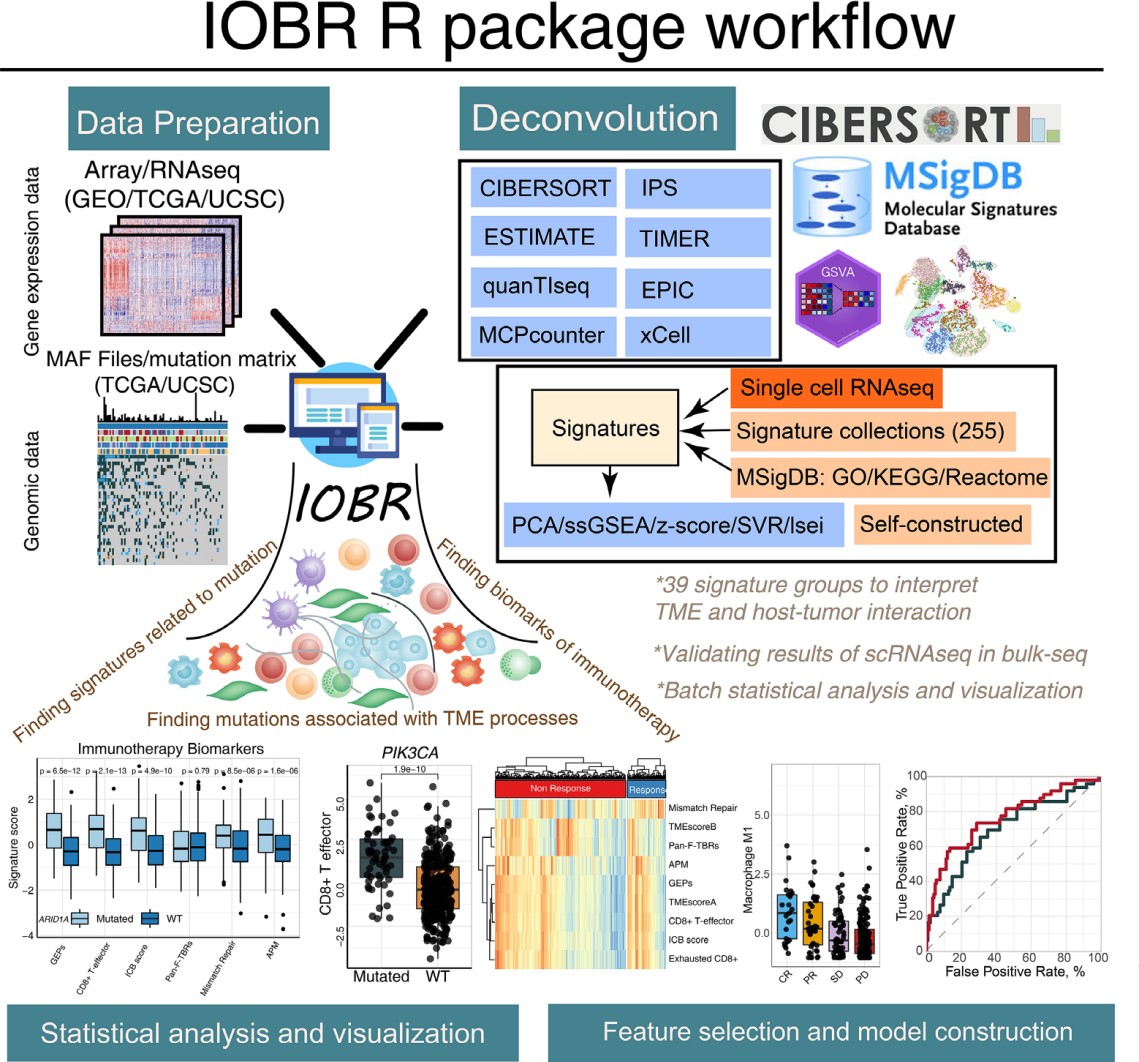
The graphical abstract outlines the workflow of the IOBR package. The IOBR R package contains corresponding data preparation, multiple deconvolution algorithms for the decoded signature estimation, TME contexture, batch statistical analyses and visualization, as well as feature selection and model construction.

**Figure 2:**
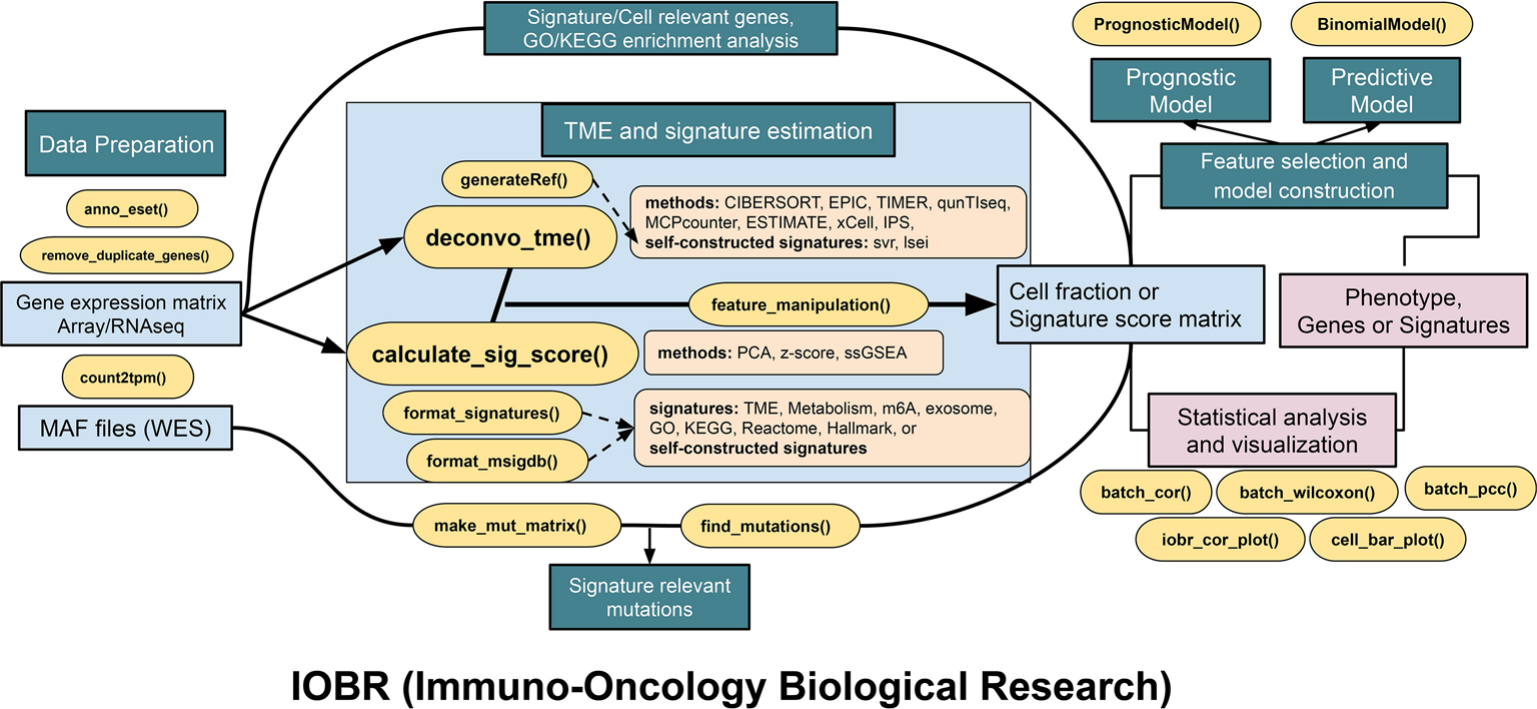
The pipeline diagram depicts functions of four analytic modules contained in IOBR. In addition to the functions for data preprocessing, the function modules comprise the following: (1) analyses of signatures pertinent to clinical phenotype, lncRNA, and targeted signatures constructed based on bulk RNA-seq or scRNA-seq data and TME deconvolution; (2) identification of phenotype relevant signatures, cell fraction, or signature genes, as well as corresponding batch statistical analyses and visualization; (3) an estimation of the specific mutation landscape associated with the signature of interest; and (4) model construction following feature selection.

IOBR comprises four function modules, namely, the signature and TME deconvolution module, the phenotype module, the mutation module, and the model construction module. The schematic workflow and functional codes are illustrated in **Figures 1**, **2**, respectively. Corresponding figures were dynamically generated following inputting function-specific parameters of pertinent modules. Details of these four modules are illustrated in the *Materials and Methods* sections. Charts derived from IOBR reach quality requirements of publication and can be flexibly modified locally. The workflow and functions of IOBR are delineated below with real-world data of the IMvigor210 cohort (7).

### Identifying TME Compositions and Signatures Relevant to Therapeutic Response

**Figure 3:**
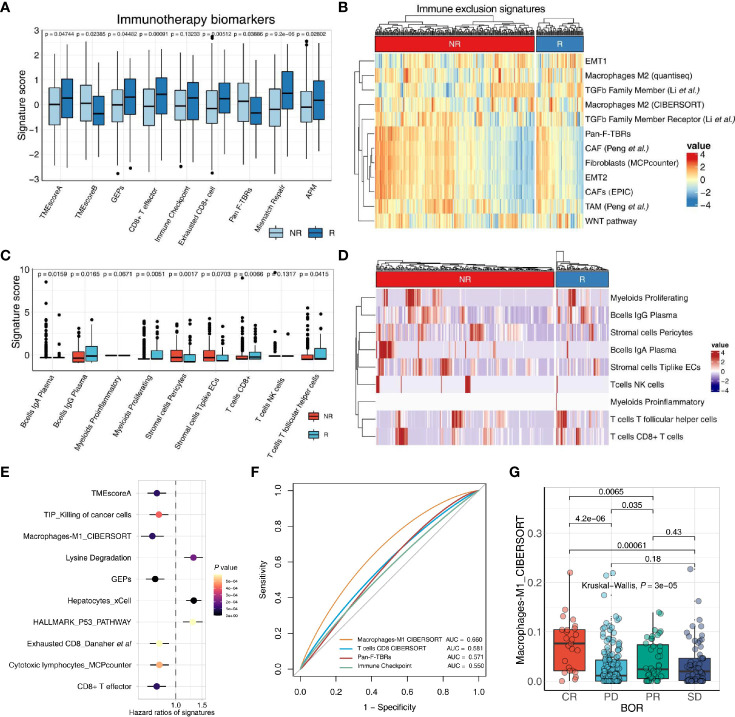
IOBR deciphers TME components and phenotype related signatures derived from bulk-seq and scRNA-seq data. **(A, B)** The boxplot **(A)** and heatmap **(B)** delineate the putative biomarkers and TME cell signatures enrolled in IOBR, and identities signatures associated with immunotherapy best overall response, by implementing IOBR to dissect bulk RNA-seq data of patients in the IMvigor210 bladder cancer cohort. APM, antigen-processing machinery; NR, non-responder; R, responder. **(C, D)** The boxplot **(C)** and heatmap **(D)** leveraging specific cell-type gene expression signatures generated from prior single-cell analysis to decipher the TME landscape of bulk-seq transcriptomic and clinical data in the IMvigor210 cohorts. The patients were classified by the responses to immune checkpoint inhibition and TME compositions statistically associated with treatment sensitivity. Subsequently, the patients were enumerated and batch visualized. **(E, F)** IOBR dissected the association between signatures and clinical phenotypes in the IMvigor210 bladder cancer cohort. **(E)** The forest plot with hazard ratios of multiple signatures, integrated a list of survival analysis outputs. **(F)** The ROC curve delineated multiple signatures for predicting the immunotherapy response, displayed in the order of their corresponding AUC (Area Under Curve) for effective comparison. **(G)** The boxplot evaluated the correlation between the macrophage M1 infiltration and the therapy response which is a category variable. The statistical discrepancy of the macrophage M1 infiltration between any categories was analyzed using the Kruskal–Wallis test and exhibited in the boxplot.

In comparison to most of the methods which calculate a single signature with a specific methodology after execution, IOBR exhibited good performance in delineating immune-tumor crosstalk by detecting a succession of gene signatures published or generated by users at the same time with multiple methodology options. Herein, we further demonstrate the utility of IOBR by deciphering the TME landscape in the increasingly accumulating bulk RNA-seq data (**Figures 3A, B**).

The transcriptomic and matched clinical data derived from patients with metastatic urothelial cancer who underwent anti-PD-L1 immunotherapy (atezolizumab) (7) are available for download at http://research-pub.gene.com/IMvigor210CoreBiologies. RNA-seq count data were transformed into TPM to estimate multiple gene signature collected in IOBR through *count2tpm* and *calculate_sig_score* functions. Moreover, the *remove_batcheffect* function for removing the batch effects across datasets when dissect tumor microenvironment is built based on the *ComBat* function derived from the *sva* R package. The output of *remove_batcheffect* could be visualized as a plot with all samples grouped using the PCA method. Further, the applicability of IOBR in bulk RNA-seq studies to batch analyze and display putative signatures related to therapy response using the *iobr_cor_plot* function consistently corroborated prior reports. Estimation of biomarkers for treatment sensitivity and signature defining TME compositions could be depicted with a boxplot or a heatmap.

Collectively, IOBR tremendously simplified the TME dissection and signature calculation analysis procedure and enriched corresponding outputs of multi-omics studies. Incorporating the immuno-oncology analyses pipelines, to some extent, may promisingly lower the bioinformatical threshold for understanding complex TME-tumor interaction to unseal tumor-host interplay mechanisms, serving optimized cancer treatment. The *remove_batcheffect* function for removing the batch effects across datasets when dissect tumor microenvironment, which is based on “*ComBat*” function derived from “*sva*” R package.

### Leveraging Signatures Generated From scRNA-seq Analyses to Decipher bulk-seq Data

Currently, single-cell analyses have revealed a vast heterogeneity of intratumoral cell states at an unprecedentedly high resolution. However, the technical simplicity and low-cost places bulk RNA-seq data remains to be the primary method of gene expression determination and gene signature estimation. With assistance from IOBR, users could leverage the merit of signatures derived from single-cell analysis to dissect tumor heterogeneity using bulk RNA-seq data.

We utilized scRNA-seq data derived from patients in a colon rectal cancer cohort to extract cell-type-specific gene expression signatures that were identified by cluster analysis in the literature (22). Marker genes of each cell type are identified by differential expression analysis. Here, based on the prior knowledge of cell-type-specific gene expression signatures, we could decipher the bulk RNA-seq data of the IMvigor210 cohort (7) by using IOBR to implement the linear svr algorithm of CIBERSORT or lsei algorithm (9). Batch visualization of TME compositions associated with immunotherapy best overall response were quickly output and offered several display options, such as boxplots and heatmaps (**Figures 3C, D**). Notably, IOBR is amenable to enumerate the TME populations with high accuracy for corroborating the discovery of single-cell studies or to uncover novel clinical transitions in bulk RNA-seq settings, translating the oncoming wealth of single-cell sequencing research into biological insight.

### Batch Analysis and Visualization

Moreover, based on the simplified analyses procedure, the *iobr_cor_plot* function is included into IOBR to facilitate its implement for quick exploration of multiple data. The *iobr_cor_plot* function dynamically generates statistical results and efficaciously depicts the correlation between signatures and targeted phenotype, such as therapeutic responses and carcinogenic infection statuses. The *sig_forest* function facilitates users to integrate the survival analysis output originated from the *batch_survival* function, and depicts a forest plot with hazard ratios of multiple signatures (**Figure 3F**). Moreover, leveraging signature to predict a specific phenotype, is a well-recognized method in preclinical bioinformatic analysis. The function *sig_roc* based on pROC R package is capable of delineating AUC curves of multiple signatures (**Figure 3G**). The parameter *compare_method* in this function enables users to compare the statistical difference between any two signatures of interest with an optional method. The *sig_box* function could be employed to infer the correlation between a category variable and a specific signature, with a boxplot displaying the statistical discrepancy of the signature score between any categories (**Figure 3E**).

Additionally, IOBR is capable of rapidly visualizing the relationships between signature genes and the targeted variable (binary or continuous) with identical methods. Likewise, IOBR is also feasible to identify signatures significantly correlated with a signature of interest. Notably, the *iobr_cor_plot* function is also available to define the signatures correlated with lncRNA profiling, by extracting targeted gene from the lncRNA expression matrix as a phenotype. The subsequent batch correlation analysis procedure is described similarly above. Furthermore, considering the fact that multiple signatures and signature genes could be enriched, IOBR enrolls a subset of functions for batch statistical analysis and visualization. IOBR comprises the batch survival analysis for either continuous signature scores or categorized phenotype subgroups, and aforementioned batch correlation analysis uses statistical tests including Wilcoxon test and Partial correlation coefficient (PCC).

### Evaluating Mutations Associated With Specific Signatures and Delineating Pertinent Mutation Landscapes

Elucidating the TME, genomic alteration landscapes, and deciphering the latent correlation would provide insight which could optimize patient stratification and therapeutic intervention. The caveat that specific somatic gene mutations could drive tumorigenesis, altering the vulnerability of cancer cells to anti-tumor immune cells and immunotherapy in multiple cancers should be recognized. To illustrate these functions, we have taken the IMvigor210 cohort and performed effective integrated analysis of genomic and transcriptomic data using the IOBR tool.

**Figure 4:**
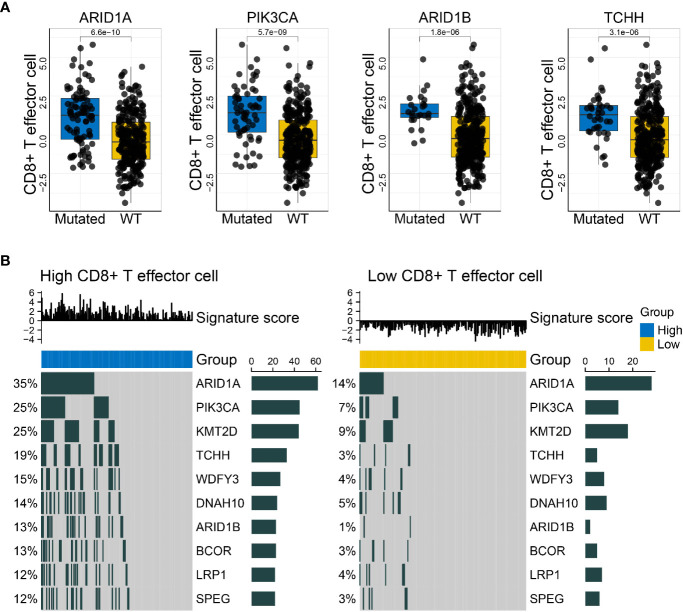
IOBR delineates the mutations correlated with the CD8^+^T effector signature and the corresponding oncoplot. **(A)** Boxplots displayed the mutations significantly associated with CD8^+^T effector signature expression, comprising *PIK3CA*, *ARID1A*, *ARID1B*, and *TCHH* (*p* = 5.7 × 10^−09^, 6.6 × 10^−10^, 1.8 × 10^−06^, and 3.1 × 10^−06^, respectively). The blue and yellow colors represent mutated and wild-type statuses. Each dot displays a patient within the IMvigor210 cohort in pertinent subgroups. **(B)** Oncoprints depicted the genomic alteration landscapes in the context of high- and low- CD8^+^T effector signature score. The upregulation of *ARID1A*, *PIK3CA*, *KMT2D*, *TCHH*, *WDFY3*, *DNAH10*, *ARIDB1*, *BCOR*, *LRP1*, and *SPEG* were observed in the high CD8^+^T effector expression setting. The numbers on the left green bars and on the right side collectively demonstrated the mutation frequency of each gene.

The *make_mut_matrix* function in IOBR was implemented *via* inputting the genomic data in MAF format, which generated an output file amenable for the *find_mutations* function. Subsequent execution of the *find_mutations* function also acquired both the genomic MAF data and the gene signature matrix of interest. Given the well-recognized significance of CD8^+^ T cell in anti-tumor immunity, we focused on the CD8^+^ T effector signature whose detail estimation was introduced in Case Study 1. Thereafter, we obtain a succession of mutations associated with the CD8^+^ T effector signature, and visualized the discrepancy of CD8^+^ T effector signature score in wildtype and mutated settings, including the mutation states of *PIK3CA*, *ARID1A*, *ARID1B*, and *TCHH* (**Figure 4A**). Alternatively, the results could be efficiently displayed with an oncoplot showing the genomic alteration landscape in high- and low-CD8^+^ T effector score subsets respective of their comparisons (**Figure 4B**). A remarkable increase in mutation frequency, including the aforementioned genes, was observed in the oncoplots.

Overall, the integration of genomic and transcriptomic data may renew and deepen our understanding of tumor progression and provide therapeutic insight by broadly taking into consideration of the crosstalk between the genome, metabolism, and TME profiles. The IOBR tool is capable of substantially simplifying these analyses procedures.

## Discussion

The complexity and increasing accumulation of multi-omics datasets pose new opportunities and challenges for integrative analysis of immuno-oncology by requiring simplification of the interpretation without sacrificing accuracy. Our study developed a comprehensive computational tool, IOBR, to dissect host-tumor interaction and signatures for therapeutic sensitivity. Four major analytic modules were provided, allowing effective and systematical analysis of tumor immunologic, clinical, genomics, and scRNA-seq data.

With this current era of immunotherapy and big data, identifying novel biomarkers and calculating signatures to finetune therapy strategies have come to the spotlight of immune-oncology. In addition to systematic estimation of published signature scores and signatures constructed by users, IOBR is able to find and interpret: lncRNA profiles, gene alteration landscapes, and scRNA-seq results. Notably, the validation of signatures generated by single-cell analysis is also involved, which relies intensely on large bulk RNA-seq datasets. Additionally, the model construction module potentiates the innovative clinical translation of signatures genes into prediction of tumor prognosis, therapy response, and tumor resistance. Moreover, the TME is an essential constituent of tumor immunity, and the correlation between TME heterogenicity and clinical phenotype is pivotal for preclinical oncology research. The IOBR R package offers multiple available deconvolution methods that removed the roadblock for decoding TME contexture. TIMER is a published web tool integrating six algorithms for inferring immune cell composition from bulk tumor transcriptome profiles (23). However, despite the convenience of intuitive outputs provided by TIMER 2.0, the upload of large datasets proves challenging for a web-based tool, an issue that could be tackled using R package tools to better analyze data with a larger volume of samples and to conveniently acquire large datasets.

With the multi-omics data accumulation, we anticipate IOBR will attract broad application in immuno-oncology and facilitate the accelerated discovery of latent immune evasion mechanisms leading to the discovery of novel therapeutic targets. IOBR represents a contribution to the computational toolbox for unveiling immune-tumor interactions from multi-omics data, being implemented in preclinical research of tumor heterogeneity and plasticity, and being instrumental in providing the impetus for precision immunotherapy.

## Data Availability Statement

The original contributions presented in the study are included in the article/**Supplementary Material**. Further inquiries can be directed to the corresponding author.

## Author Contributions

Study concept and design: DZ. Acquisition of data: ZY, JW, WQ, NH, and LS. Analysis and interpretation of data: DZ and RZ. Package development: DZ, ZY, and RS. Drafting of the tutorial: DZ, ZY, and RS. Drafting of the manuscript: ZY and DZ. Critical revision of the manuscript for important intellectual content: GY, MS, XL, and WL. Obtaining funding: XL and WL. Administrative, technical, or material support: YX, JB, and YL. Supervision: GY and WL. Shiny application development: YX. All authors contributed to the article and approved the submitted version.

## Funding

This work was supported by the National Natural Science Foundation of China (Nos. 81772580, 81472594 and 81770781), the Guangzhou Planned Project of Science and Technology (No. 201803010070), the Guangdong-Macao Science and Technology Innovation Joint Fund Project and Hong Kong-Macao Science and Technology Achievement Transformation Project in Guangdong (No. 2020A0505090007).

## Conflict of Interest

The authors declare that the research was conducted in the absence of any commercial or financial relationships that could be construed as a potential conflict of interest.

## Supplementary Material

Supplementary Table 1 Multiple signatures included in IOBR. Collection and pertinent citations of all 255 signatures enrolled in the IOBR R package.

Supplementary Table 2 The signatures collected in IOBR were systematically categorized into 39 groups. The categories contain TME cell populations (classified either by deconvolution methods or cell types), signatures of immunophenotype, tumor metabolism, hypoxia, and EMT.
